# Clean Milk Film

**Published:** 1920-04-17

**Authors:** 


### Clean Milk Film.

The National Clean Milk Society, in furtherance of their propaganda for raising the hygienic standard of milk and milk products, propose to initiate British dairy farmers and the public into the methods by which New York has become an example in safeguarding the purity of its milk supplies. This has been attempted by means of lectures and demonstrations, and now the aid of the film is being invoked. The film was shown last week for the first time at a private view at the Polytechnic Cinema, in the presence of a company that included Mr. W. Buckley, the Controller of Milk Supplies, Ministry of Food.

The Society has spared the feelings of the audience—or spectators should it be?—by presenting an example of how milk should be produced and distributed, rather than by giving examples of some of the unhygienic methods and conditions which are only too common. Cows are shown being washed and groomed and milked on the farm, then the milk being received for analysis in the depot, whence it is sent, cooled and in sterilised cans, to the receiving centre in New York. Here the milk is Pasteurised, cooled, and bottled by automatic machinery. The bottles travel along a moving platform, and are filled with milk as they go. The froth is drawn out, the disc adjusted, and then the cap is put on, and the bottle with its contents is ready for delivery to the consumer. In New York, as in a number of other American cities, it is illegal to deliver milk except in sealed bottles. It is also illegal to refill the bottles elsewhere than on the retailers' premises.

One of the morals sought to be enforced by the film is that it is quite impossible for the small retailer to handle milk efficiently. There ought, it is insisted, to be large organsations suitably equipped. This, of course, is a question of considerable controversy as to the methods by which it should be put into effect, but it ought somehow to be done.

The Government have undertaken to bring in a Milk Amendment Bill, to provide, among other things, for the issuing of revocable licences to milk sellers, and for the continuation and expansion of the system of grading milk already introduced by the Food Controller. In the view of the Society, these powers, together with those of the Milk and Dairies Act, which will be brought into operation when the amending Bill has become law, should provide a basis for the improvement of the country's milk supply.

